# Differential Sleep Traits Have No Causal Effect on Inflammatory Bowel Diseases: A Mendelian Randomization Study

**DOI:** 10.3389/fphar.2021.763649

**Published:** 2021-11-30

**Authors:** Min Chen, Wen-Yan Peng, Tai-Chun Tang, Hui Zheng

**Affiliations:** ^1^ Department of Colorectal Diseases, Hospital of Chengdu University of Traditional Chinese Medicine, Chengdu, China; ^2^ The Third Hospital/Acupuncture and Tuina School, Chengdu University of Traditional Chinese Medicine, Chengdu, China

**Keywords:** sleep disorders, inflammatory bowel disease, Mendelian randomization, drug repurposing, pathogenesis

## Abstract

**Background:** Previous studies suggested an association of sleep disorders with inflammatory bowel disease (IBD) and indicated that using pharmacological treatments for the modulation of circadian rhythms might prevent IBD pathogenesis or aggravation, but whether the effect of sleep traits on IBD was causal is inconclusive and, therefore, prevents drug repurposing based on the previous studies. We aimed to examine the causal effect of different sleep traits on the pathogenesis of IBD.

**Methods:** Genetic instruments for sleep traits were selected from the largest GWAS studies available in the UK Biobank (*n* = 449,734) and the 23andMe Research (*n* = 541,333). A two-sample Mendelian randomization (MR) study was conducted to examine the association of the genetic instruments with IBD (12,882 cases and 21,770 controls), ulcerative colitis (6,968 cases, 20,464 controls), and Crohn’s disease (5,956 cases and 14,927 controls). We applied the inverse-variance weighted (IVW) method to estimate causal effects, and we used the weighted median and MR-Egger method for sensitivity analyses.

**Results:** We found that sleep duration (OR, 1.00, 95% CI 1.00–1.01), short sleep duration (OR, 1.07, 95% CI 0.41–2.83), morningness (OR, 1.05, 95% CI 0.87–1.27), daytime napping (OR, 1.64, 95% CI 0.62–4.4), frequent insomnia (OR, 1.17, 95% CI 0.8–1.72), any insomnia (OR, 1.17, 95% CI 0.69–1.97), and snoring (OR, 0.31, 95% CI 0.06–1.54) had no causal effect on IBD, and these sleep traits had no causal effect on ulcerative colitis and Crohn’s disease either. Most of the sensitivity analyses showed consistent results with those of the IVW method.

**Conclusion:** Our MR study did not support the causal effect of sleep traits on IBD. Pharmacological modulation of circadian rhythms for the prevention of IBD pathogenesis was unwarranted.

## Introduction

Inflammatory bowel disease (IBD) is a chronic, autoimmune disease of the gastrointestinal tract, including two subtypes—Crohn’s disease (CD) and ulcerative colitis (UC). The pathogenesis of IBD includes genetic predisposition, environmental risk factors, and impaired immune activation (Ho et al., 2019; Ramos and Papadakis, 2019). IBD has affected 6.8 million people worldwide as of 2017 (Alatab et al., 2020). The annual cost of treatment per patient exceeds $25,000, which brings heavy health economic burden (Kaplan, 2015). Patients with IBD were more prone to develop colorectal cancer (CRC) than healthy controls, which makes IBD a major health problem and serious health concern (Hendriksen et al., 1985; Ng et al., 2017).

Sleep disorders affect both innate and adaptive immune function, which is gradually recognized as one of the potential environmental triggers of IBD (Ananthakrishnan, 2015; Rozich et al., 2020). The three major proinflammatory cytokines—interleukin-1β, TNF-α, and interleukin-6—are closely associated with increased non-rapid eye movement sleep, and these pro-inflammatory cytokines are also important in the pathogenesis of IBS (Ali and Orr, 2014; Qazi and Farraye, 2019). Animal studies showed that sleep disorders promoted colonic inflammation and worsened the severity of colitis (Preuss et al., 2008; Tang et al., 2009). Clinical studies showed a correlation of sleep disorders with IBD, in which a significant decrease in sleeping time and sleeping efficiency was closely related to the development and disease activity of IBD (Chakradeo et al., 2018; Chrobak et al., 2018; Jarasvaraparn et al., 2019; Marinelli et al., 2020). A recent systematic review and meta-analysis confirmed the association between poor sleep quality and increased risk of IBD activity (Hao et al., 2020). However, whether sleep disorders have causal effects on IBD pathogenesis is still uncertain, since the relationship between IBD and sleep disorders might be bidirectional. In addition, previous studies were conducted with observational designs, which were prone to confounding issues and unable to clarify a cause-and-effect relationship. Besides, the uncertain causal effect of sleep disorders on IBD, which of the sleep traits—sleep duration, chronotype, and insomnia—will have a more significant impact on the pathogenesis of IBD, has not been fully elucidated. Most studies investigating the role of sleep disorders on IBD pathogenesis focused on estimating overall sleep quality (e.g., by using the Pittsburgh Sleep Quality Index) or one of the sleep traits (Hao et al., 2020; Orr et al., 2020; Conley et al., 2021; Leal et al., 2021). Uncovering the causality of differential sleep traits on IBD might offer new insights into the pathogenesis of IBD and inform future intervention studies.

Mendelian randomization (MR) analyses used genetic instruments to infer a cause-and-effect relationship between exposures and outcomes. Genetic variants and allelic randomization minimize the problem of confounding issues and reverse causation, which provides stronger evidence than traditional observational studies in inferring cause–effect relationships (Davies et al., 2018). Therefore, we conducted an MR study to examine whether differential sleep traits had causal effects on IBD pathogenesis and determine which of the sleep traits have a more significant impact.

## Methods

### Study Design

We performed a two-sample MR study, using summary-level data from publicly available genome-wide association studies (GWAS). The data of the GWAS studies were obtained from UK Biobank and 23andMe Research. The study design and reporting conformed to STROBE-MR (Davey Smith et al., 2019).

### Ethics

Our analysis used published studies or publicly available GWAS summary data. No original data were collected for this manuscript, and thus, no ethical committee approval was required. Each study included was approved by their institutional ethics review committees, and all participants provided written informed consent.

### Data Sources

Table 1 shows a summary of the study populations, the number of genetic instruments—single nucleotide polymorphisms (SNPs)—for the sleep traits, and the heritability or variance explained in the sleep traits. Ethical approval was acquired from each database.

**TABLE 1:** Characteristics of the study population.

Traits	Source	Sample size	Ancestry	Number of SNPs	Female (%)	Age (SD)	Adjusted covariates	Heritability or variance explained in the traits
Sleep duration	UK Biobank Dashti et al. (2019)	446,118	European	78	NA	40–69 years	Age, sex, the first 10 PCs, and genotype array	The SNP-based heritability was 9.8% (SE 0.1%), and the SNPs explained 0.69% of the variance in sleep duration.
Short sleep duration (<7 h)	UK Biobank Dashti et al. (2019)	411,934 (106,192 cases and 305,742 controls)	European	27	NA	40–69 years	Age, sex, the first 10 PCs, and genotype array	The SNP-based heritability was 7.9% (SE 0.1%).
Morningness	UK Biobank Jones et al. (2019)	403,195 (252,287 cases and 150,908 controls)	European	153	45.7	56.8 (8.0)	Age, sex, study center, and genotyping release	The SNP-based heritability was 13.7% (95% CI 13.3–14.0).
Morningness	23andMe Research Hu et al. (2016); Jones et al. (2019)	248,098 (120,478 cases and 127,622 controls)	97% European	83	48.4	22.6%, 30–45 years; 31.6%, 45–60 years; 36.3%, >60 years	Age, sex	The SNPs explained 21% (95% CI 13–29) of the variance in morningness.
Daytime napping	UK Biobank Wang et al. (2019)	452,071 (104,786 cases and 347,285 controls)	European	42	54.2	60.19 (7.38) versus 56.19 (8.06)	Age, sex, the first 10 PCs, and genotype array	The SNP-based heritability was 11.9% (SE 0.1%), and the SNPs explained 1.1% of the variance in daytime napping.
Daytime napping	23andMe Research Wang et al. (2019); Dashti et al. (2021)	541,333 (274,062 cases and 267,271 controls)	European	19	NA	NA	Age, sex, the first 4 PCs, and genotyping platforms	NA
Frequent insomnia	UK Biobank Lane et al. (2019)	237,627 (129,270 cases and 108,357 controls)	European	49	61.5% versus 42.2%	57.69 (7.5) versus 55.49 (8.39)	Age, sex, the first 10 PCs, genotype array, and genetic correlation matrix	The SNP-based heritability was 16.7%.
Any insomnia	UK Biobank Lane et al. (2019)	453,379 (345,022 cases and 108,357 controls)	European	29	56 versus 42.2%	56.87 (8.01) versus 55.49 (8.39)	Age, sex, the first 10 PCs, genotype array, and genetic correlation matrix	NA
Snoring	UK Biobank Campos et al. (2020)	408,317 (151,077 cases and 257240 controls)	European	41	40.74 versus 61.44%	57.01 (7.7) versus 56.6 (8.21)	Age, sex, the first 20 PCs, and genotype array	The SNP-based heritability was 9.9 (0.39) %.
Inflammatory bowel diseases	IBD Genetics Consortium Liu et al. (2015)	34,652 (12882 cases and 21770 controls)	European	27	53.5	31.25(NA)	15 PCs chosen from the first 20 PCs	NA
Ulcerative colitis	IBD Genetics Consortium Liu et al. (2015)	27,432 (6968 cases and 20464 controls)	European	27	47.9	34.1 (15.78)	7 PCs chosen from the first 20 PCs	NA
Crohn’s disease	IBD Genetics Consortium Liu et al. (2015)	20,883 (5956 cases and 14927 controls)	European	27	54.9	28.39 (14.16)	10 PCs chosen from the first 20 PCs	NA

PCs, principal components. SD, standard deviation. SE, standard error. and SNP, single-nucleotide polymorphisms.

### Sleep Duration

Genetic instruments of self-reported sleep duration were obtained from a GWAS dataset containing 446,118 participants from the UK Biobank (Dashti et al., 2019). The sleep duration data were acquired by asking participants as to how many hours of sleep do they get in every 24 h? The total duration was calculated as a continuous variable, and two categorical variables—short (<7 h) and long (>9 h) duration—were computed. Participants who reported extremely short (<3 h) or long (>18 h) sleep duration were excluded. The GWAS study compared the group of short duration (*n* = 106,192) with the reference group (sleep duration between 7 and 9 h, *n* = 305742), and the group of long sleep duration (*n* = 34,184) was also compared with the reference group. The SNPs for long sleep duration were not included in our analysis, since the number of SNPs for long sleep duration was small, and they were under the risk of weak instrument bias (Dashti et al., 2019).

### Morningness

Morningness was defined as the characteristic of an individual being the most active and alert during the morning. Genetic instruments of morningness were obtained from two GWAS datasets—403,195 participants from the UK Biobank and 248,098 participants from 23andMe Research (Hu et al., 2016; Jones et al., 2019). The morningness in the UK Biobank population was determined by a question—“Do you consider yourself to be?” Participants who answered “definitely a morning person” or “more a morning than evening person” were treated as participants with the characteristics of morningness. Participants in the 23andMe research dataset were invited to answer surveys online, on which the participants were asked whether they were naturally a night person or morning person (Hu et al., 2016).

### Daytime Napping

Daytime napping is referred to an irrepressible sleep pattern in a day. Genetic variants indicative of daytime napping were obtained from the UK Biobank (*n* = 452,071) and 23andMe Research (*n* = 541,333) (Wang et al., 2019; Dashti et al., 2021). The participants were asked a question—“Do you take a nap during the day?” 38.2 and 5.3% of participants answered “sometimes” and “always”, respectively, in the UK Biobank population; and 43 and 7.6% reported “sometimes” and “always”, respectively, in the 23andMe Research.

### Insomnia

Genetic instruments were obtained in a GWAS study in the UK Biobank population (*n* = 453,379), in which the participants were asked “Do you have trouble falling asleep at night or do you wake up in the middle of the night?” The participants who responded “usually” were classified as frequent insomniacs (*n* = 129,270), and those who responded “usually” or “sometimes” were classified as non-insomniacs (*n* = 345,022) (Lane et al., 2019).

### Snoring

Genetic variants that correlated with snoring were acquired from the UK Biobank population (*n* = 408,317), in which the participants were asked “Does your partner or a close relative or friend complain about your snoring?” with response options of “Yes”, “No”, “Don’t know”, or “Prefer not to answer”. The participants who answered “Don’t know” or “Prefer not to answer” were excluded from the dataset.

### Inflammatory Bowel Diseases

Genetic variants for IBD were acquired from a GWAS study from the IBD Genetics Consortium (Liu et al., 2015). The GWAS study included 5956 participants with CD, 6968 participants with UC, and 21,770 controls to discover the genetic variants.

### Selection of Genetic Instruments

Genetic instruments should be associated with the sleep traits (the relevance assumption of MR). To ensure the relevance assumption, the correlation *p*-value should be less than 5 × 10^8^ in the corresponding GWAS study. To ensure that there are no unmeasured confounders of the association between genetic instruments and IBD (the independence assumption), we excluded the single nucleotide polymorphisms (SNPs) with r^2^—a measurement of the pairwise-linkage disequilibrium—larger than 0.001.

### Statistical Analysis

The Wald ratio, obtained by dividing the SNP-outcome association estimate by the SNP-exposure association estimate, was used to evaluate the size of the causal effect of sleep traits on IBD. The ratio estimates (β values) for the SNPs were then pooled by using inverse-variance weighted (IVW) meta-analysis; and the pooled β values and corresponding 95% confidence intervals (95% CIs) were calculated through the meta-analysis. The β values were transformed into odds ratios (ORs) using the formula—β = ln (OR). Scatter plots of SNP-outcome associations versus SNP-exposure associations were provided for the MR analysis.

Heterogeneity of the IVW meta-analysis was measured by Cochran’s Q test and *I* ^*2*^ statistics, and an *I* ^*2*^ value smaller than 40% was considered an indication of unimportant heterogeneity—as stated by the Cochrane Handbook (Higgins, 2011). To evaluate the strength of the included IVs, we calculated the mean F-statistics for the sleep traits.

Several sensitivity analyses were performed by using the methods of the simple median, weighted median, and MR-Egger regression. The median-based methods (simple median and weighted median) have greater robustness than the inverse-variance weighted and MR-Egger methods when individual genetic variants with strongly outlying causal estimates are included. The pooled ratio estimates from the simple median method were obtained by computing the ratio estimates from each genetic variant and finding the median estimate; while the estimates from the weighted median method were obtained by computing normalized inverse-variance weights for each genetic variant and pooling the estimates with the incorporation of the acquired weights. The MR-Egger method allows one or more genetic variants to have pleiotropic effects. The ratio estimates from the MR-Egger method were obtained by regression of the ratios of the SNP-outcome associations versus the SNP-exposure associations, with the weights being the inverse-variances of the associations with the outcome.

To evaluate the potential pleiotropy in the IVW model, we performed the Mendelian Randomization Pleiotropy RESidual Sum and Outlier (MR-PRESSO) analysis. To validate the independent causal effects of sleep traits on IBD, we performed a multivariable MR analysis. To evaluate whether the study results were affected by confounding bias, we performed linkage disequilibrium score (LDSC) regression analysis; an LDSC intercept larger than 1.3 indicated that the results might be affected by confounding bias (Bulik-Sullivan B. K. et al., 2015). In addition, cross-trait LDSC analysis was performed to determine the genetic correlation between the traits, and the SNP genetic correlation estimates and their standard errors (SEs) were reported (Bulik-Sullivan B. et al., 2015).

## Results

**FIGURE 1:**
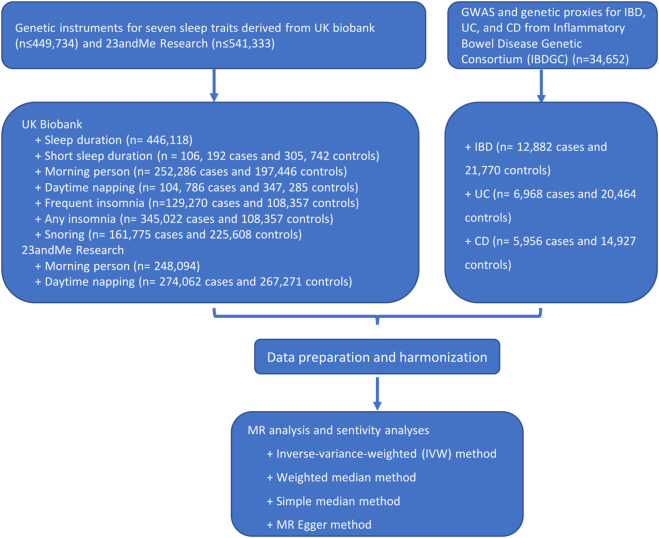
Flowchart of the study process. Abbreviations: GWAS, genome-wide association studies. IVW, inverse-variance weighted. MR, Mendelian randomization. Annotation: UK Biobank is a large-scale biomedical database and research resource, containing in-depth genetic and health information from half a million UK participants. The 23andMe cohort is one of the largest re-contactable research databases of genotypic and phenotypic information.

**FIGURE 2:**
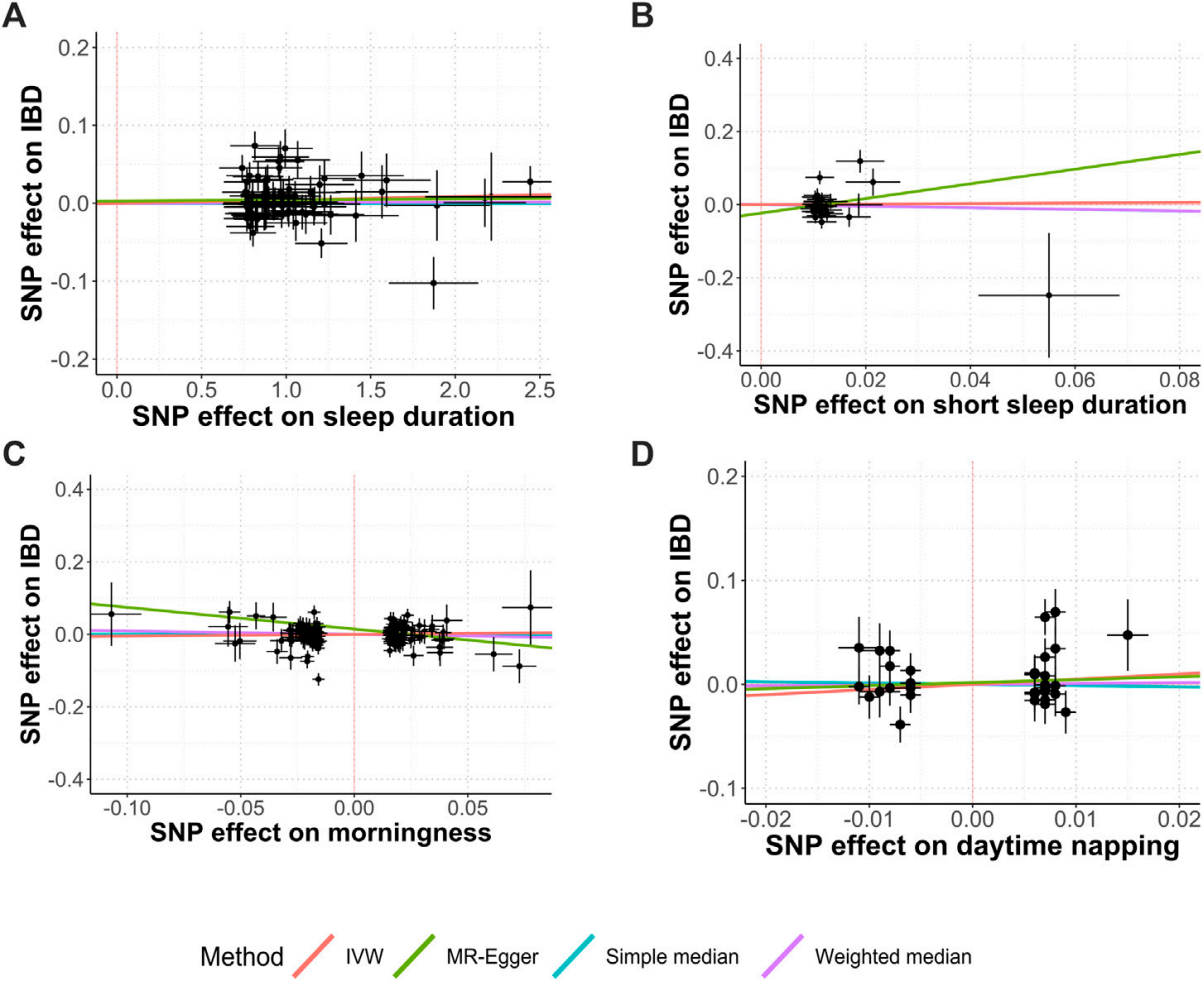
Causal relationships between sleep duration, morningness, daytime napping, and IBD. Abbreviations: IVW, inverse-variance weighted. MR, Mendelian randomization. SNP, single-nucleotide polymorphisms. Annotation: Scatter plots of the IBD-SNP associations (y-axis) versus the sleep-traits-SNP associations (x-axis) were showed, with horizontal and vertical lines showing 95% confidence intervals for each association. **(A)** Sleep duration; **(B)** Short sleep duration; **(C)** Morningness; **(D)** Daytime napping.

**FIGURE 3:**
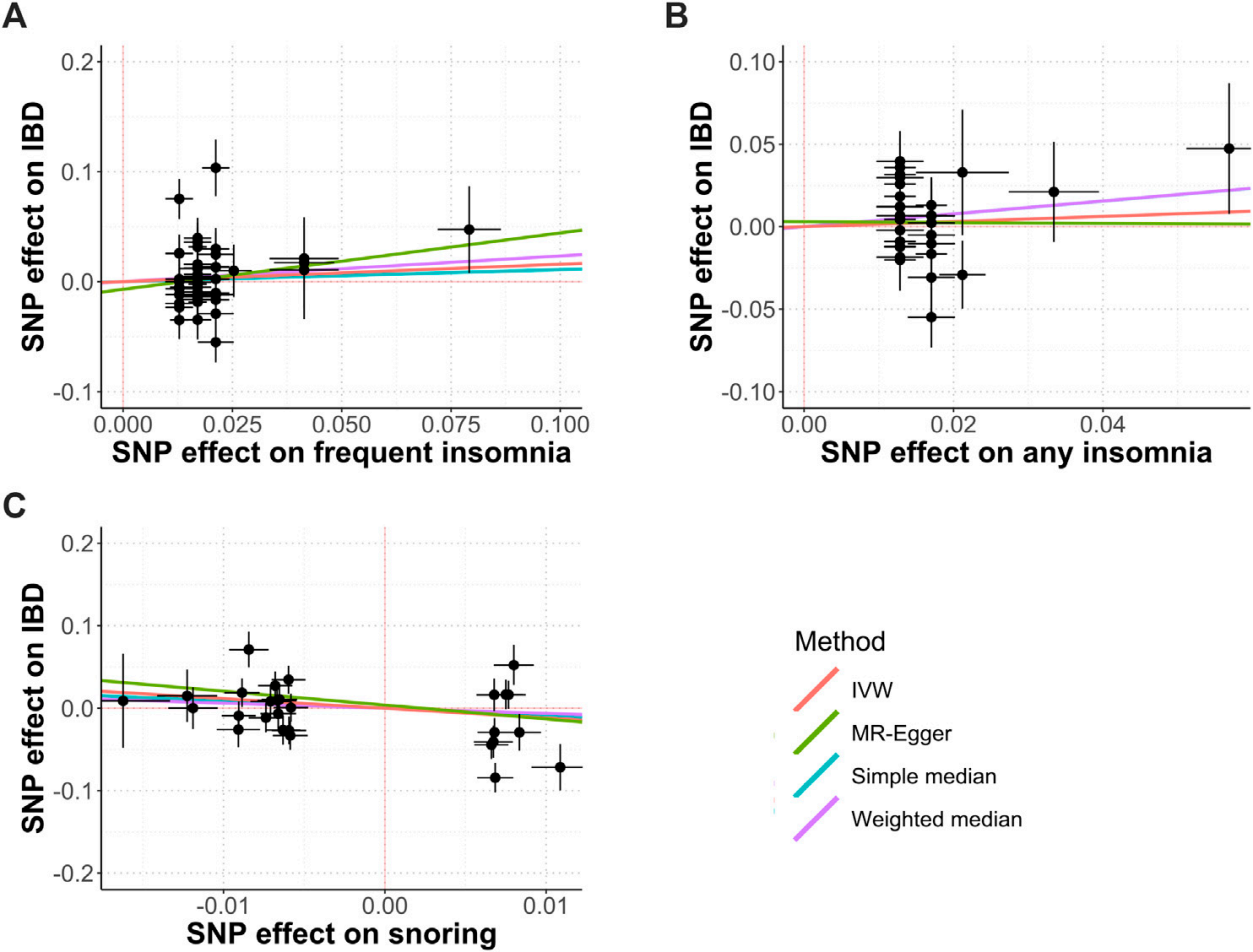
Causal relationships between insomnia, snoring, and IBD. Abbreviations: IVW, inverse-variance weighted. MR, mendelian randomization. SNP, single nucleotide polymorphisms. Annotation: Scatter plots of the IBD-SNP associations (y-axis) versus the sleep-traits-SNP associations (x-axis) were showed, with horizontal and vertical lines showing 95% confidence intervals for each association. **(A)** Frequent insomnia; **(B)** Any insomnia; **(C)** Snoring.

**FIGURE 4:**
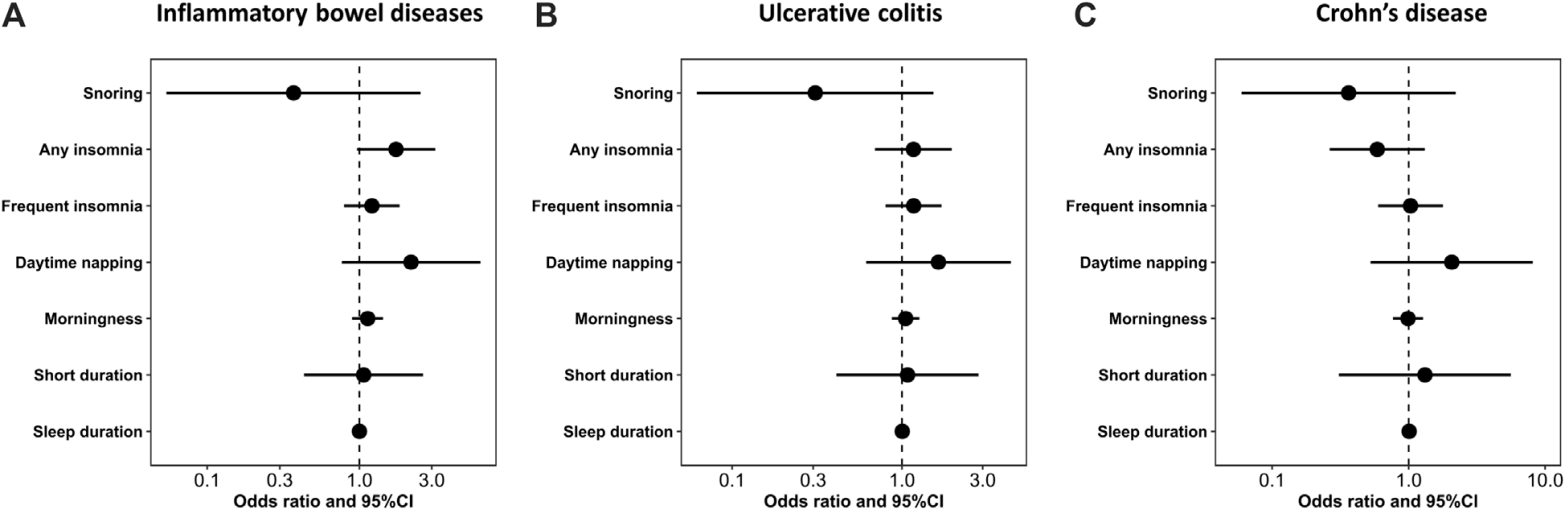
The size of the causal effect of differential sleep traits on IBD. Abbreviations: 95%CI, 95% confidence interval. Annotation: The forest plots showed the size of the causal effect of differential sleep traits on IBD. The dots were the pooled effects—measured by odds ratio—of the sleep traits, and the horizontal lines were the corresponding 95%CIs. When the 95%CIs contained the null value (odd ratio = 1), there was no causal effect of sleep traits on IBD.

Figure 1 shows the study process and data sources. Figures 2, 3 and Supplementary Figures S1, S2 show the scatter plots of SNP-outcome associations versus SNP-exposure associations (IVW analysis and sensitivity analyses). Figure 4 shows the causal effects of differential sleep traits on IBD, which was estimated by IVW methods. Supplementary Table S1 shows the results of sensitivity analyses of the causal effects. The results of the analysis calculated by using the 23andMe Research are shown in Supplementary Figures S5, S6. The detailed information of the instrumental variants is provided in Supplementary Tables S2–S8.

### Sleep Duration and IBD

The IVW analysis showed that sleep duration had no causal effect on IBD (OR, 1.00; 95% CI, 1.00 to 1.01; Cochran’s Q = 122.33; *I* ^*2*^ = 37.9%; mean F-statistic = 1.636), UC (OR, 1.00; 95% CI, 0.99 to 1.01; Cochran’s Q = 94.18; *I* ^*2*^ = 19.3%; mean F-statistic = 1.224), or CD (OR, 1.01; 95% CI 1.00 to 1.02; Cochran’s Q = 94.18; *I* ^*2*^ = 19.3%; mean F-statistic = 1.947). A further analysis studying the causal effect of short sleep duration showed similar results (Figures 2A,B; Figure 4; Supplementary Figures S1, S2). Sensitivity analyses showed similar results (Supplementary Table S1), and the MR-PRESSO analysis revealed significant distortion in the causal estimates (Supplementary Table S9). The LDSC analysis showed a regression intercept of 1.218 (residual standard error 1.197).

### Morningness and IBD

The IVW analysis showed that morningness had no causal effect on IBD (OR, 1.05; 95% CI, 0.87 to 1.27; Cochran’s Q = 238.37; *I* ^*2*^ = 37.1%; mean F-statistic = 1.582), UC (OR, 1.13; 95% CI, 0.9 to 1.44; Cochran’s Q = 241.46; *I* ^*2*^ = 37.9%; mean F-statistic = 1.61), or CD (OR, 0.99; 95% CI 0.77 to 1.27; Cochran’s Q = 236.7; *I* ^*2*^ = 36.6%; mean F-statistic = 2.797) (Figure 2C, Figure 4; Supplementary Figures S1, S2). The sensitivity analyses showed similar results (Supplementary Table S1), and the MR-PRESSO analysis revealed significant distortion in the causal estimates (Supplementary Table S9). The analysis of the 23andMe Research dataset did not support a causal effect of morningness on IBD and UC (Supplementary Figures S3, S4). However, it showed a causal effect of morningness on CD (OR, 0.87; 95% CI, 0.76 to 0.99; *p* = 0.037; mean F-statistic = 1.254). The LDSC analysis showed a regression intercept of 1.656 (residual standard error 1.347).

### Daytime Napping and IBD

The IVW analysis showed that daytime napping had no causal effect on IBD (OR, 1.65; 95% CI, 0.62 to 4.40; Cochran’s Q = 48.45; *I* ^*2*^ = 27.8%; mean F-statistic = 1.384), UC (OR, 2.19; 95% CI, 0.77 to 6.27; Cochran’s Q = 34.59; *I* ^*2*^ = 0%; mean F-statistic = 1.02), or CD (OR, 2.07; 95% CI 0.53 to 8.13; Cochran’s Q = 50.54; *I* ^*2*^ = 30.8%; mean F-statistic = 1.447; Figure 2D, Figure 4; Supplementary Figures S1, S2). The sensitivity analyses showed similar results (Supplementary Table S1), and the MR-PRESSO analysis revealed significant distortion in the causal estimates (Supplementary Table S9). The analysis of the 23andMe Research dataset did not support a causal effect of daytime napping on IBD and UC (Supplementary Figures S5, S6). However, it showed a causal effect of daytime napping on CD (OR, 6.03; 95% CI, 1.15 to 31.65; *p* = 0.034; mean F-statistic = 2.922). The LDSC analysis showed a regression intercept of 1.62 (residual standard error 1.209).

### Insomnia and IBD

The IVW analysis showed that frequent insomnia had no causal effect on IBD (OR, 1.17; 95% CI, 0.8 to 1.72; Cochran’s Q = 87.5; *I* ^*2*^ = 45.1%; mean F-statistic = 1.81), UC(OR, 1.21; 95% CI, 0.79 to 1.84; Cochran’s Q = 67.55; *I* ^*2*^ = 28.9%; mean F-statistic = 1.4), or CD (OR, 1.03; 95% CI 0.6 to 1.78; Cochran’s Q = 96.7; *I* ^*2*^ = 50.4%; mean F-statistic = 1.974; Figures 3A,B, Figure 4; Supplementary Figures S1, S2). The sensitivity analyses showed similar results (Supplementary Table S1), and the MR-PRESSO analysis revealed significant distortion in the causal estimates (Supplementary Table S9). A further analysis studying the causal effect of any kind of insomnia showed similar results (Figure 2F; Figure 3). Sensitivity analyses showed similar results, except that any kind of insomnia had a causal effect on UC and CD in the median methods (Supplementary Table S1). The LDSC analysis showed a regression intercept of 1.668 (residual standard error 1.231).

### Snoring and IBD

The IVW analysis showed that snoring had no causal effect on IBD (OR, 0.31; 95% CI, 0.06 to 1.54; Cochran’s Q = 76.12; *I* ^*2*^ = 64.5%; mean F-statistic = 2.926), UC (OR, 0.37; 95% CI, 0.05 to 2.53; Cochran’s Q = 69.34; *I* ^*2*^ = 61.1%; mean F-statistic = 2.571), or CD (OR, 0.36; 95% CI 0.06 to 2.21; Cochran’s Q = 51.43; *I* ^*2*^ = 47.5%; mean F-statistic = 1.919; Figure 3C; Figure 4; Supplementary Figures S1, S2). Sensitivity analyses showed similar results (Supplementary Table S1), and the MR-PRESSO analysis revealed significant distortion in the causal estimates (Supplementary Table S9). The LDSC analysis showed a regression intercept of 1.535 (residual standard error 1.203).

Multivariate MR analyses, for the purpose of validating the findings of the abovementioned analyses, showed similar results that differential sleep traits had no causal effects on IBD, UC, and CD (Supplementary Tables S10–S12). The cross-trait LDSC analysis demonstrated genetic correlations in sleep durations and insomnia, morningness and insomnia, daytime napping and snoring, and IBD and CD (Supplementary Table S13).

## Discussion

### Main Findings and Strength of the Study

We aimed to study whether differential sleep traits had causal effects on IBD, UC, or CD, and our IVW analysis found that the sleep traits—sleep duration, morningness, daytime napping, insomnia, and snoring—had no causal effect on the pathogenesis of IBD, UC, or CD. Most of the sensitivity analyses and the analysis based on another large dataset from 23andMe Research showed consistent results with those of the IVW analysis.

We used the method of MR studies to find out the role of sleep traits in the pathogenesis of IBD. Compared with previous observational studies that found the correlation between sleep disorders and IBD, the MR-design study is less prone to be biased by confounders. We used the largest GWAS studies to select the genetic instruments and verified the findings using another large-sample GWAS study—23andMe Research—which ensured that our study met the relevance and independence assumptions. In addition, we performed sensitivity analyses to ensure that the exclusion restriction assumption was held.

### Interpretation of the Results

Previous cohort studies found a bidirectional relationship between sleep disorders and IBD (Ananthakrishnan, 2015; Chrobak et al., 2018, 2018; Jarasvaraparn et al., 2019; Marinelli et al., 2020; Conley et al., 2021; Leal et al., 2021), and a systematic review confirmed the findings (Hao et al., 2020). In addition, a review including three clinical trials and fifteen non-clinical studies found that melatonin had a positive impact on IBD (Mozaffari and Abdollahi, 2011); this finding suggested a causal effect of sleep disorders on the pathogenesis of IBD since melatonin is commonly used to treat sleep disorders (Mozaffari and Abdollahi, 2011). The relationship between sleep disorders and abnormal immune response was recognized as the main mechanism of sleep disorders causing the pathogenesis of IBD (Eissa et al., 2020). However, the causal effect of sleep disorders on IBD was still questioned (Parekh et al., 2015), since most of the evidence came from observational studies.

Our study result did not support a causal effect of sleep disorders on the pathogenesis of IBD, which is contrary to previous studies. Sleep duration, frequent insomnia, and morningness had little effect on IBD or its subtypes. Although daytime napping and snoring seem to have a larger impact on the pathogenesis of IBD, the wide 95% CIs of their effect sizes exclude the possibility of the causal effect. It is worth noting that MR studies always have wider 95% CIs than observational studies adopting linear regression analysis with adjustment for covariates (Davies et al., 2018), so our study might have insufficient power to detect the causal effect of daytime napping or snoring. However, it should also be noted that we verified the results of daytime napping by using another dataset from 23andMe Research and still found similar results, which implied that daytime napping was unlikely to have a substantial impact on IBD pathogenesis. Regarding the fact that snoring might have a positive effect on preventing IBD, the result had no practical value and, therefore, was not warranted further studies.

Sleep disorders might be confounding factors of the causal effect of psychological disorders (anxiety and depression) on IBD—an explanation for the relationship between sleep disorders and IBD pathogenesis shown in previous studies—since the bidirectional relationship between sleep disorders and anxiety or depression is acknowledged (Fang et al., 2019). The influence of the brain–gut axis in IBD was increasingly recognized (Gracie et al., 2019). On the one hand, psychological comorbidity was prevalent in patients with IBD, with a prevalence as high as 32% (Barberio et al., 2021); and on the other hand, the presence of anxiety or depression was closely correlated to the development of new-onset IBD (Gracie et al., 2019). Basic science also showed that the increased level of stress led to increased catecholamine secretion and enlarged sympathetic outflow that caused proinflammatory effects on the gastrointestinal system, and mast cells and macrophages were stimulated and activated by inflammatory cytokines (Gracie et al., 2019). In addition, a growing body of evidence showed that antidepressants could lower IBD activity and relieve bowel symptoms (Mikocka-Walus et al., 2020), which suggested a brain-to-gut regulation and, therefore, a causal effect of psychological disorders on IBD. However, owing to a lack of experimental evidence, this hypothesis has not been tested, which warrants future studies.

### Generalizability

Our study adopted genetic data from the two study populations that were of European ancestry, which ensured the genetic homogeneity and robustness of the study results. However, it limits the generalizability of the study findings to other populations. Sleep disorders might have causal effects on the pathogenesis of IBD in other populations, and our study did not test the results in other populations because of lack of relevant data. It should also be noted that SNPs associated with the IBD outcome were determined based on the diagnosis and presence of IBD, but not on disease progression or disease severity. Accordingly, whether sleep disorders have impacts on the progression of IBD (leading to escalation of pharmacological treatments, disease relapse, or surgery) is still unknown, and our study results could not be generalized to guide whether an aggressive treatment for sleep disorders is needed for patients with IBD to prevent disease progression.

### Limitations

Owing to the two-sample MR design, we used summary-level statistics instead of individual-level statistics. However, regarding the fact that the included data were adjusted for similar characteristics (i.e., age, sex, and body mass index) and that the two-sample MR design is less susceptible to weak instrument bias (Lawlor, 2016), we speculated that our study result was robust. We did not perform a bidirectional MR, since the causal effect of IBD on sleep is deducible and confirmed with solid evidence (Rozich et al., 2020). In addition, the results of mean F-statistics indicated a risk of bias in weak instruments, which warrants further studies to confirm our study results.

## Conclusion

Our study did not support a causal effect of differential sleep traits on the pathogenesis of IBD, and the result could be robust because of the large sample size of the study, multiple sensitivity analyses, and verification in two GWAS study populations of sleep traits. Future studies focusing on the causal effect of psychological disorders on IBD pathogenesis are warranted, as well as future studies conducted in populations other than European ancestry.

## Data Availability

The original contributions presented in the study are included in the article/Supplementary Material, and further inquiries can be directed to the corresponding author.

## Abbreviations

95% CIs, 95% confidence intervals; CD, Crohn’s disease; CRC, colorectal cancer; GWAS, genome-wide association studies; IBD, inflammatory bowel disease; IVW, inverse-variance weighted; MR, Mendelian randomization; OR, odds ratio; SNPs, single nucleotide polymorphisms; UC, ulcerative colitis.
