# Supplementary material for: Differential Sleep Traits Have No Causal Effect on Inflammatory Bowel Diseases: A Mendelian Randomization Study
Source: Front Pharmacol. 2021 Nov 30;12:763649. doi: 10.3389/fphar.2021.763649 (PMC8669049; doi:10.3389/fphar.2021.763649)
Supplement: Supplementary file 1 [file DataSheet1.docx]

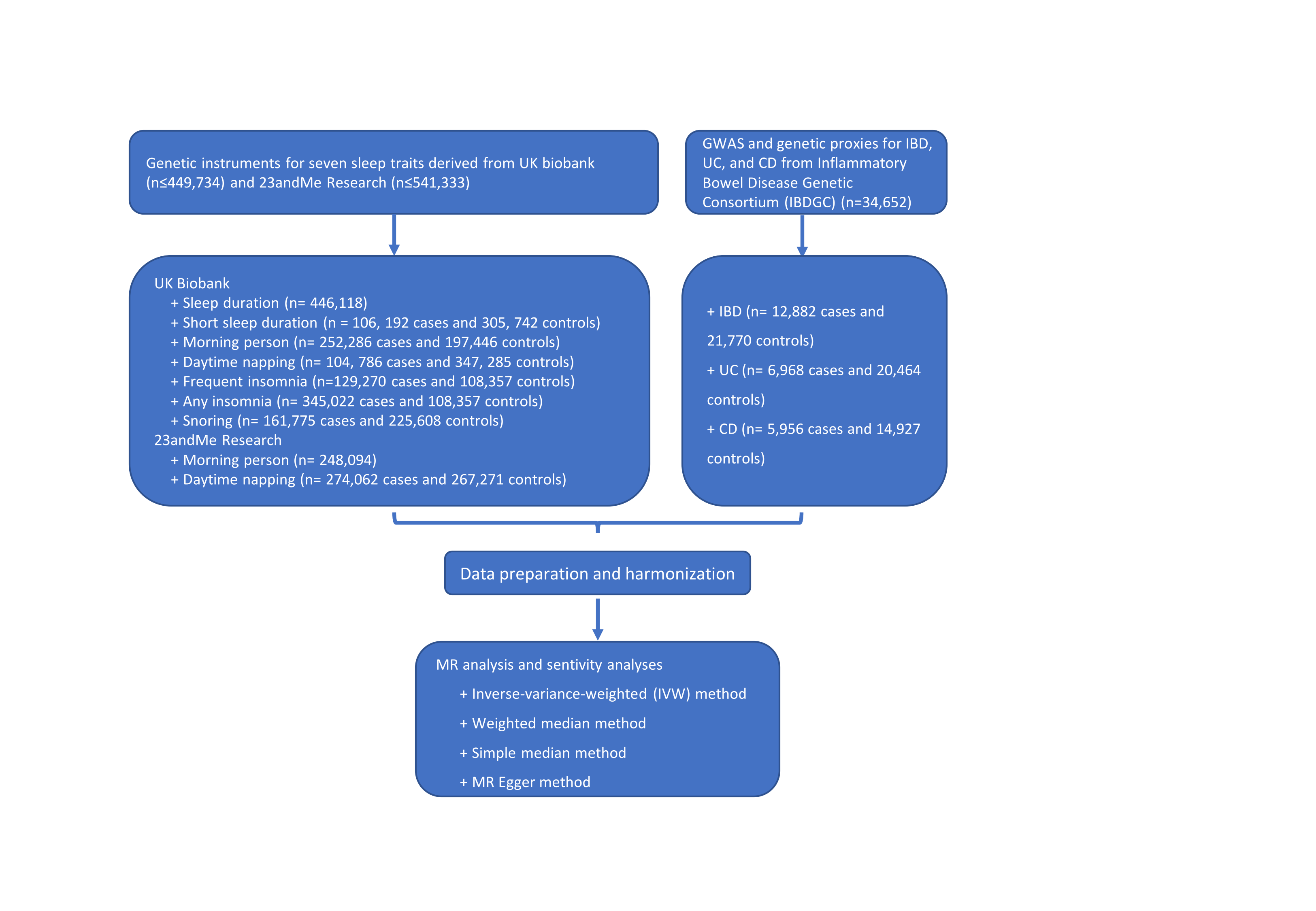


**Figure 1. Flowchart of the study process**

Abbreviations: GWAS, genome-wide association studies. IVW, inverse-variance weighted. MR, mendelian randomization.

Annotation: UK Biobank is a large-scale biomedical database and research resource, containing in-depth genetic and health information from half a million UK participants. The 23andMe cohort is one of the largest re-contactable research databases of genotypic and phenotypic information.


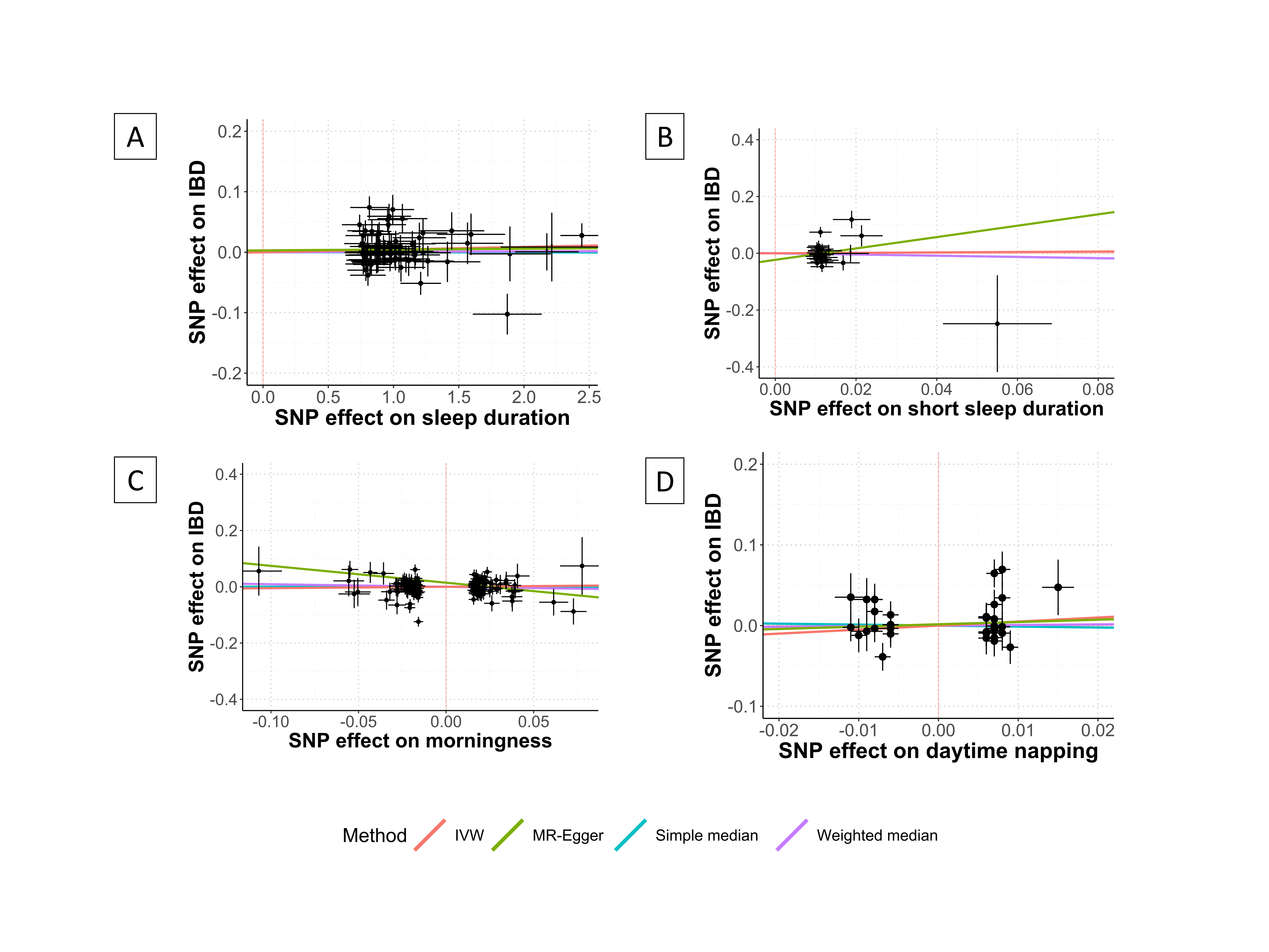


**Figure 2. Causal relationships between sleep duration, morningness, daytime napping, and IBD**

Abbreviations: IVW, inverse-variance weighted. MR, mendelian randomization. SNP, single nucleotide polymorphisms.

Annotation: Scatter plots of the IBD-SNP associations (y-axis) versus the sleep-traits-SNP associations (x-axis) were showed, with horizontal and vertical lines showing 95% confidence intervals for each association. (A) Sleep duration; (B) Short sleep duration; (C) Morningness; (D) Daytime napping.


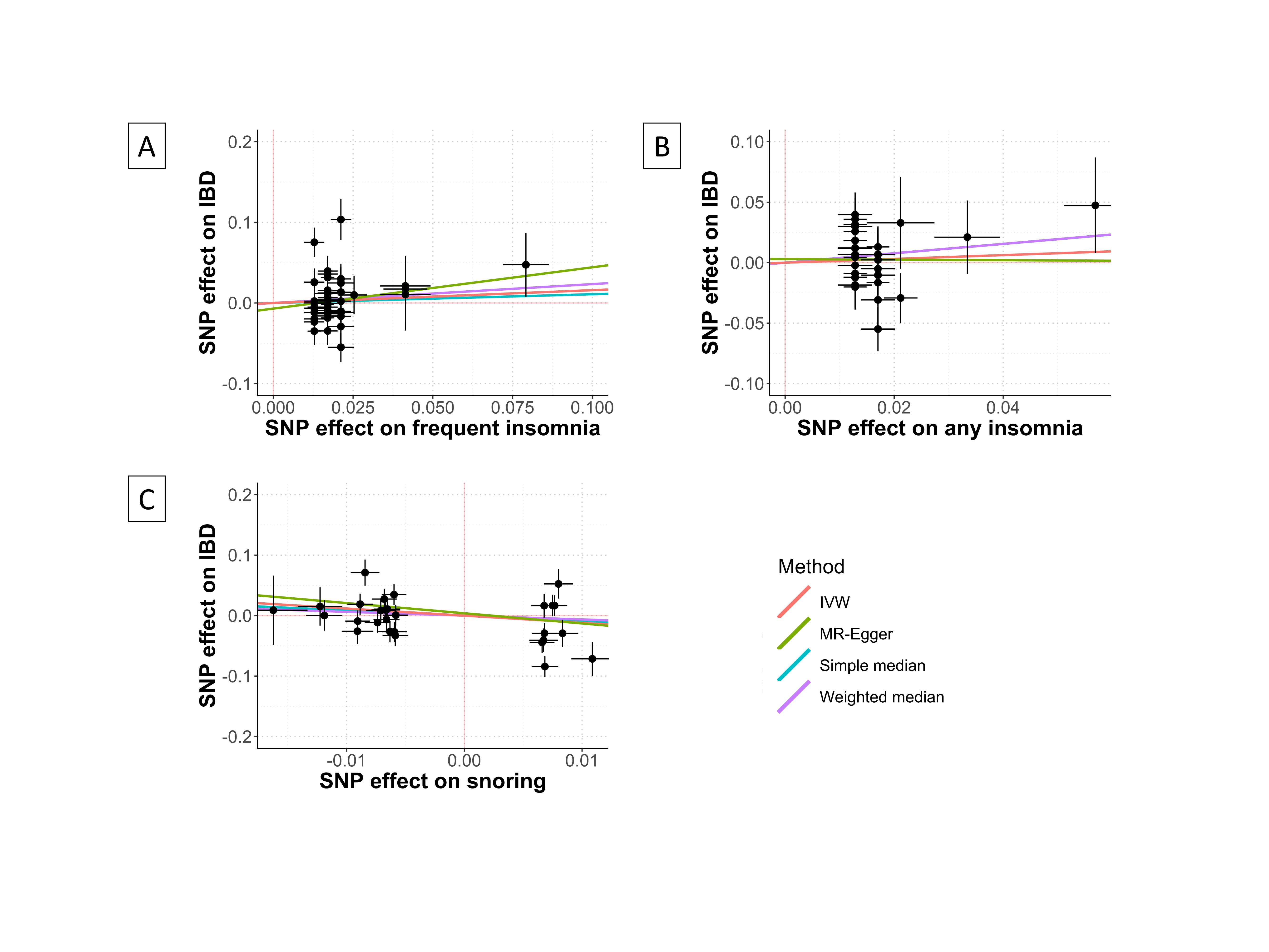


**Figure 3. Causal relationships between insomnia, snoring, and IBD**

Abbreviations: IVW, inverse-variance weighted. MR, mendelian randomization. SNP, single nucleotide polymorphisms.

Annotation: Scatter plots of the IBD-SNP associations (y-axis) versus the sleep-traits-SNP associations (x-axis) were showed, with horizontal and vertical lines showing 95% confidence intervals for each association. (A) Frequent insomnia; (B) Any insomnia; (C) Snoring.


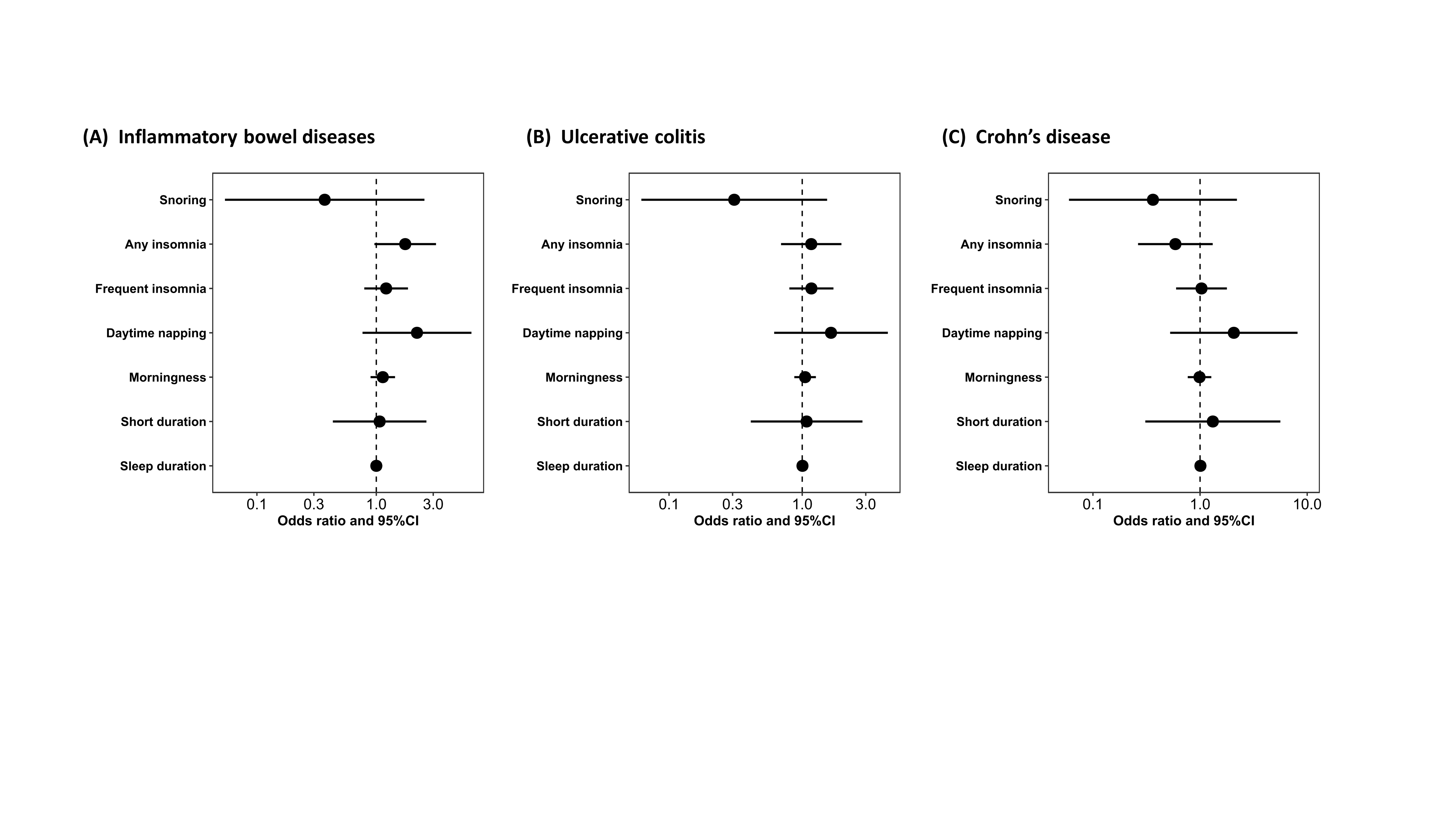


**Figure 4. The size of the causal effect of differential sleep traits on IBD**

Abbreviations: 95%CI, 95% confidence interval.

Annotation: The forest plots showed the size of the causal effect of differential sleep traits on IBD. The dots were the pooled effects—measured by odds ratio—of the sleep traits, and the horizontal lines were the corresponding 95%CIs. When the 95%CIs contained the null value (odd ratio = 1), there was no causal effect of sleep traits on IBD.
